# Pesticides Exposure-Induced Changes in Brain Metabolome: Implications in the Pathogenesis of Neurodegenerative Disorders

**DOI:** 10.1007/s12640-022-00534-2

**Published:** 2022-07-04

**Authors:** Joel Arvin Rodrigues, Rekha K. Narasimhamurthy, Manjunath B. Joshi, Herman Sunil Dsouza, Kamalesh Dattaram Mumbrekar

**Affiliations:** 1 Manipal School of Life Sciences, Manipal Academy of Higher Education, Manipal, Karnataka India 576104; 2 Department of Radiation Biology and Toxicology, Manipal School of Life Sciences, Manipal Academy of Higher Education, Manipal, Karnataka India 576104; 3 Department of Ageing Research, Manipal School of Life Sciences, Manipal Academy of Higher Education, Manipal, Karnataka India 576104

**Keywords:** Neurodegenerative disorders, Brain, Pesticides, Metabolites, Neurotoxicity, Organophosphate

## Abstract

Pesticides have been used in agriculture, public health programs, and pharmaceuticals for many decades. Though pesticides primarily target pests by affecting their nervous system and causing other lethal effects, these chemical entities also exert toxic effects in inadvertently exposed humans through inhalation or ingestion. Mounting pieces of evidence from cellular, animal, and clinical studies indicate that pesticide-exposed models display metabolite alterations of pathways involved in neurodegenerative diseases. Hence, identifying common key metabolites/metabolic pathways between pesticide-induced metabolic reprogramming and neurodegenerative diseases is necessary to understand the etiology of pesticides in the rise of neurodegenerative disorders. The present review provides an overview of specific metabolic pathways, including tryptophan metabolism, glutathione metabolism, dopamine metabolism, energy metabolism, mitochondrial dysfunction, fatty acids, and lipid metabolism that are specifically altered in response to pesticides. Furthermore, we discuss how these metabolite alterations are linked to the pathogenesis of neurodegenerative diseases and to identify novel biomarkers for targeted therapeutic approaches.

## Introduction

With a sharp increase in global life expectancy, the world’s population over 60 years is expected to double from 12 to 22% between 2015 and 2050 (WHO 2021). The rise in the proportion of the aging population has led to an increase in age-related neurodegenerative disorders. Various metabolic pathways like fatty acid β-oxidation pathway, oxidative stress, mitochondrial dysfunction, glycerophospholipid metabolism, tryptophan metabolism, and glutathione metabolism have been shown to play a role in neurodegenerative diseases (Widner et al. 2002; Jenner 2003; Smeyne and Smeyne 2013; Bose and Beal 2016; Yan et al. 2021). The causative factors contributing to the onset and development of complex and multigenic neurodegenerative diseases have been attributed to genetic and environmental factors (Brown et al. 2005; Cannon and Greenamyre 2013; Pihlstrøm et al. 2017).

Among the environmental factors, pesticides are one of the many factors linked to the onset of neurodegenerative disorders (Chin-Chan et al. 2015). Pesticide exposure occurs through either occupational exposure, including pesticide applicators, mixers, pesticide distributors (Maroni et al. 2006), dietary routes, or drinking water (Lewis et al. 1988). This might lead to delayed toxic manifestation at low-level and high-level exposure scenarios. Pesticides intended to eradicate agricultural pests by targeting the nervous system of pests may also directly target the nervous tissue of other organisms with similar neurochemical processes (Keifer and Fireston 2007; Bjørling-Poulsen et al. 2008). Pesticides disrupt critical cellular mechanisms that sustain the metabolic requirements and activity of the nervous system (Keifer and Fireston 2007). Studies speculate that most Alzheimer’s disease (AD) and Parkinson’s disease (PD) cases observed in the older population might have been exposed to pesticides long before diagnosis (de Pedro-Cuesta et al. 2015; Yan et al. 2021). Furthermore, Freire and Koifman (2012) reviewed several published prospective and case–control studies and found evidence of an association between pesticide exposure and PD (Freire and Koifman 2012).

Metabolomics allows monitoring the changes in the whole metabolome, reflecting the context-dependent variation in genomic, transcriptomic, and proteomic fluctuations. However, metabolomics does not reveal the cause of the disease but shows the final results of normal or altered metabolic functions. Metabolomic assessment of exposure to different classes of pesticides such as organophosphates (OPs), organochlorines (OCs), and pyrethroids (PYRs) in biofluids has shown alteration in metabolites related to oxidative stress, inflammatory reactions, and mitochondrial dysfunction (Yan et al. 2021). Furthermore, it has been noted that many of the metabolic pathways altered by pesticides are common to neurodegeneration (Kori et al. 2016; Yan et al. 2021). So, metabolomics can detect metabolite level deviation and identify the affected metabolic pathways, which helps determine the etiology of a neurodegenerative disorder (Jové et al. 2014). Furthermore, diagnostic markers are unavailable for early detection of neurological syndromes such as AD, PD, and amyotrophic lateral sclerosis (ALS); therefore metabolomics is now slowly gaining importance in biomarker identification. The present review provides an overview of specific metabolic pathways, including tryptophan metabolism, glutathione metabolism, dopamine metabolism, energy metabolism, mitochondrial dysfunction, fatty acids, and lipid metabolism altered in response to pesticides and their link to neurodegenerative disorders.

## Methodology

**Fig. 1:**
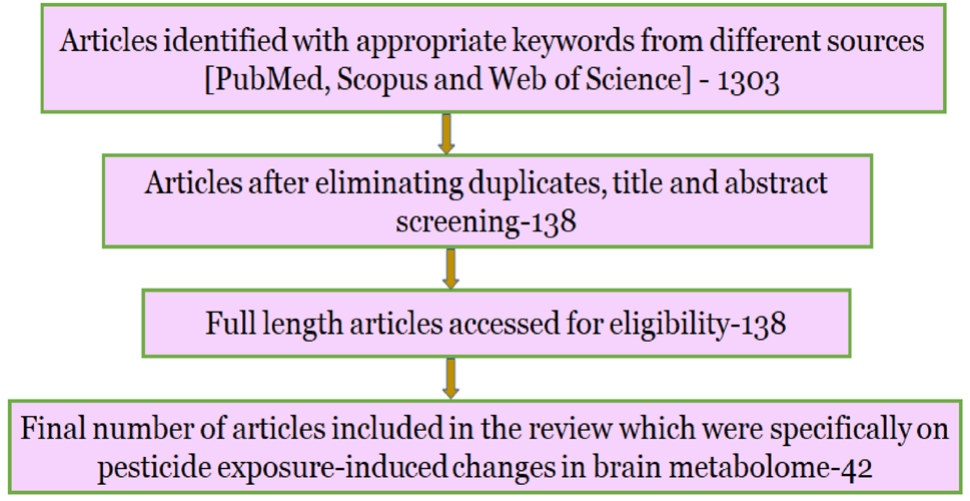
Flowchart depicting the literature search strategy for the review

For the present review, we scoured the online scientific databases like Pubmed, Scopus, and Web of Science with a set of keywords pertaining to the topic of the review. The keywords like “pesticides, neurodegeneration, metabolomics,” “pesticides, neurotoxicity, metabolomics,” “pesticides, hippocampus, neurodegeneration,” and “pesticides, mitochondria, metabolism, brain” were used in different combinations in order to obtain studies showing alterations in different metabolic pathways. Studies conducted between 1970 and 2022, including both in vivo and in vitro studies, were considered. Only studies reporting changes in the metabolomic profile of the brain or metabolites affecting the brain were included. The web-based reviewing tool Rayyan (Ouzzani et al. 2016) was used to import and screen all the search results as per our inclusion criteria. A total of 42 studies that looked into the metabolomic changes induced by pesticides in different experimental models were found and included in the study (Fig. 1). Furthermore, we looked for studies where these metabolic changes are associated with neurodegeneration.

## Specific Metabolomic Changes

### Amino Acid Metabolism: Tryptophan Metabolism

In Tryptophan (TRT) metabolism, enzymes tryptophan 2,3-dioxygenase and the indoleamine 2,3-dioxygenase, metabolizes L-tryptophan into its metabolite L-kynurenine (KYN) (Vamos et al. 2009). KYN is further metabolized into either kynurenic acid (KYNA) or quinolinic acid (QUINA) (Sas et al. 2007; Zádori et al. 2009). KYNA binds to excitatory amino acid receptors such as N-methyl-D-aspartate (NMDA) receptors, kainite receptors, quisqualate and α-amino-3-hydroxy-5-methyl-4-isoxazolepropionic acid (AMPA) receptors (Prescott et al. 2006; Rózsa et al. 2008). NMDA receptors are induced by QUINA, causing glutamate excitotoxicity and inhibiting glutamate reuptake (Tavares et al. 2002). Lipid peroxidation and free radical production are promoted by QUINA, promoting neurotoxicity (Rios and Santamaria 1991; Behan et al. 1999). A study conducted on 22 PD patients and 11 age-matched controls displayed a higher kynurenine/tryptophan ratio (kyn/trp ratio), indicating that altered kynurenine pathway affecting TRT metabolism plays a role in PD (Widner et al. 2002).

Pyrethroids are a class of pesticides that cause modulation of sodium channel activity. When adult male Wistar rats were exposed to deltamethrin and fenpropathrin, the metabolite KYNA production was decreased by 31% (Zielińska et al. 2005). On dosage of mice with diazinon, plasma metabolites indicated that the levels of L-tryptophan metabolites were altered (Seifert and Pewnim 1992). As more than 40% of the brain kynurenine originates from the circulatory route (Gál and Sherman 1978), the changes in the levels affect the biosynthesis of the metabolites KYNA and QUINA (Seifert and Pewnim 1992).

**Fig. 2:**
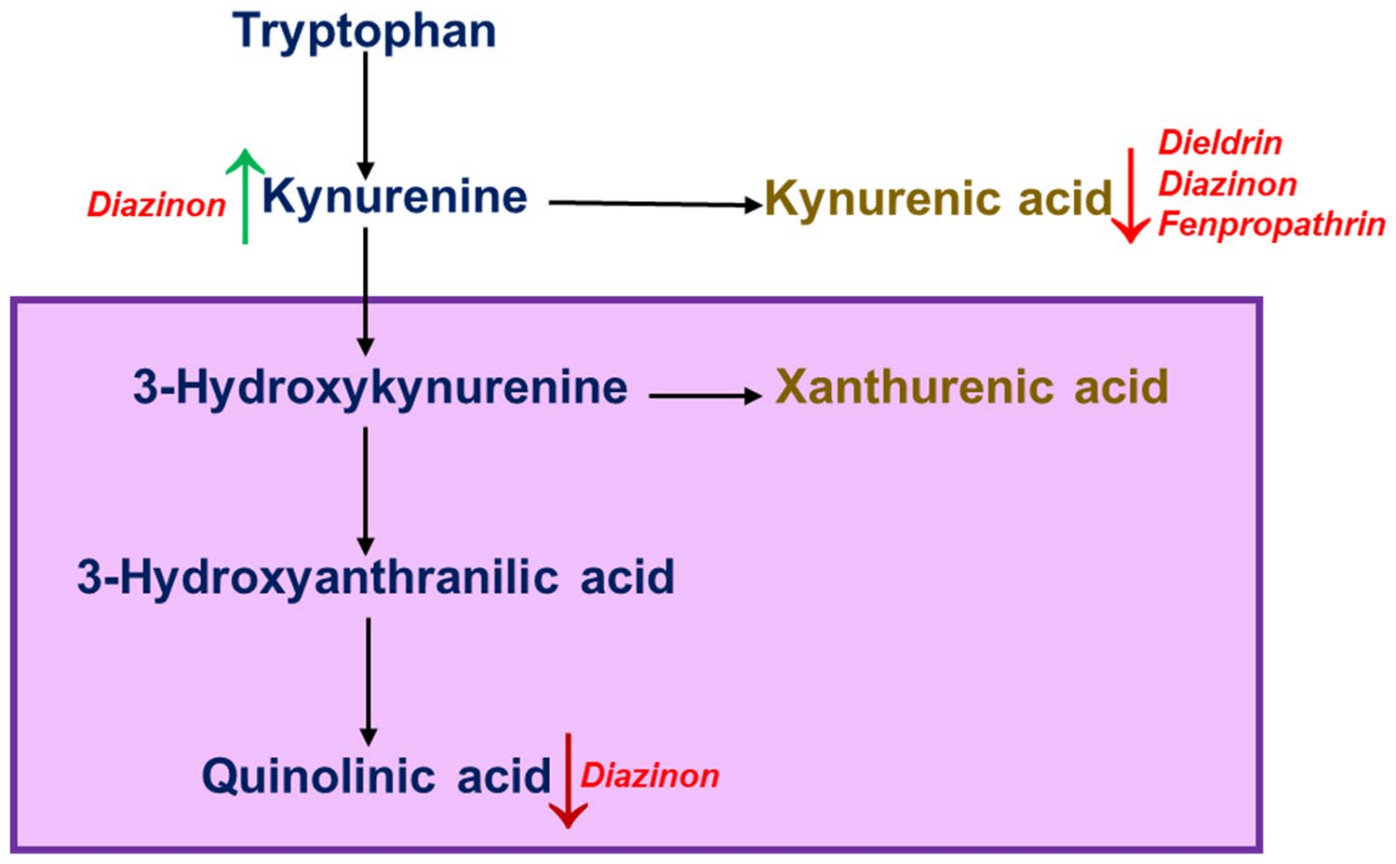
Alterations in quinolinic acid and kynurenic acid levels due to pesticide exposure

Comparing cerebrospinal fluid (CSF) levels of tryptophan in PD patients vs. healthy controls, it was determined that tryptophan levels were severely reduced in PD patients (Trupp et al. 2014). PD patients’ CSF and brain tissue showed reduced KYNA synthesis in the kynurenine pathway (Ogawa et al. 1992). Pyrethroids inhibit KYNA synthesis and tryptophan metabolism, leading to de novo generation of nicotinamide adenine dinucleotide coenzyme (NAD^+^), leading to 3-hydroxykynurenine synthesis. Studies on PD indicate that increased 3-hydroxykynurenine levels caused excitotoxicity and increased oxidative stress (Zinger et al. 2011; LeWitt et al. 2013), which are usually countered by the metabolite KYNA (Szabó et al. 2011). A metabolomics study using plasma and CSF from 20 PD patients detected reduced levels of tryptophan 3-hydroxyisovaleric acid compared to the control group leading to increased 3-hydroxykynurenine synthesis (Trupp et al. 2014). Altered tryptophan metabolism is thus a connecting link between pesticide-induced changes in metabolism and neurodegeneration. The effect of various pesticides on quinolinic acid and kynurenic acid levels is depicted in Fig. 2.

### Metabolism of Other Amino Acids

Apart from tryptophan metabolism, pesticides also drastically affect metabolism of other amino acids. Amino acids are building blocks for several other metabolic pathways, including neurotransmitter synthesis as well as purine and pyrimidine synthesis, and thus alterations in their levels can impair other necessary pathways within the brain (Rose 2019). Exposure to *p,p’*-dichloro-diphenyl-trichloroethane (DDE) led to an increase in the glutamine levels and metabolism of glutamate, aspartate, and alanine in the brain (Rodríguez-Moro et al. 2019). This pathway is the major regulator of glutamate levels and the maintenance of the homeostasis of glutamate levels through the subsequent conversion into γ-aminobutyrate (GABA), one of the main inhibitory neurotransmitters (Pardo et al. 2011). The increased levels of glutamine could be to deal with the excitotoxicity effects of the pesticide. Increased glutamine also finds its use in the purine and pyrimidine biosynthesis, Krebs, and urea cycle (Cruzat et al. 2018). Furthermore, proline, which has a prominent role in scavenging reactive oxygen species (Liang et al. 2013), was found to increase, which may indicate oxidative stress in the cell (Rodríguez-Moro et al. 2019). A group of pregnant rats exposed to a mixture of pesticides showed decreased levels of amino acids like glutamine, serine, lysine, and ethanolamine in the brain, indicating altered the amino acid metabolites (Bonvallot et al. 2018). Chlorfenapyr-treated zebrafish brain metabolome revealed a significant decrease in the metabolites like lysine, alanine, tyrosine, leucine, phenylalanine, and valine, while 7-methylxanthine and taurine were shown to be upregulated (Chen et al. 2021). Dichlorvos-poisoned broilers showed variations in amino acids like gamma-glutamylcysteine, glutathione disulfide, and dipeptide compound (Huang et al. 2022). In *Drosophila melanogaster* too, paraquat-induced reduced GABA levels with several metabolites altered in amino acid and pentose phosphate metabolism (Shukla et al. 2016).

The changes in the amino acid levels correlate with neurodegenerative conditions wherein patients of Alzheimer’s exhibited reduced levels of methionine and tryptophan along with the reduced ratio of plasma taurine with methionine and serine plasma product levels (TSM ratio); and reduced plasma tyrosine and large neutral amino acids ratio (LNAA) (Fekkes et al. 1998). Significant levels of reduction were also noted in the CSF/plasma ratio of amino acids like glutamine, alanine, phenylalanine, and valine, giving a clear indication of amino acid metabolism disruption in neurodegenerative conditions (Basun et al. 1990). Serum profiles of PD patients also displayed changes in the levels of alanine, arginine, phenylalanine, and threonine in different severity stages (Figura et al. 2018), indicating the importance of amino acid in neurodegenerative diseases.

### Glutathione Metabolism Induces Oxidative Stress

Glutathione S-transferase (GST) and reduced glutathione (GSH) systems work together to maintain redox homeostasis. In the event of increased free radicals, GST dimerizes to bind and interact with GSH and reduces the level of free radicals (Coles et al. 1988). Glutathione oxidizes to glutathione disulfide while reducing H_2_O_2_, thus reducing oxidative stress (Mishra and Srivastava 2013). An increased rate of glutathione biosynthesis is inversely correlated with decreased oxidative stress (Smeyne and Smeyne 2013), which also signals the need to remove reactive oxidative species (ROS). In neurons, approximately 85 to 90% of cellular oxygen is consumed by the mitochondria to produce energy as adenosine triphosphate molecules (ATP), resulting in the formation of ROS. Oxidative stress has been associated with PD as indicated by elevated oxidation end-products (Smeyne and Smeyne 2013).

Paraquat is known to cause Parkinson-like symptoms via oxidative and nitrosative stress (Peng et al. 2004; Shukla et al. 2014). The three pesticides, deltamethrin, glyphosate, and lambda-cyhalothrin, showed decreased GST activity (Diken et al. 2017). When male Swiss albino mice were treated with maneb and paraquat at a dose of 30 mg/kg and 10 mg/kg, their brain samples showed increased GST levels (Singhal et al. 2011). GST worked to counteract the oxidative stress caused by maneb and paraquat treatment in the nigrostriatal tissues by increasing GST levels (Patel et al. 2006). GST binding to GSH is affected due to maneb, as it mimics the properties of GSH, but due to the structural and chemical differences between maneb and GSH the normal fuctioning is inhibited (Anderson et al. 2021). Diminished GST activity in CSF (Mazzetti et al. 2015) and brain specimens (Lovell et al. 1998) has been observed in AD patients.

A group of pregnant rats exposed to a mixture of pesticides simulating the exposure scenario in Brittany in 2004 (cropland and vegetable and fruit contamination) showed decreased levels of several metabolites, including higher levels of oxidized glutathione (Bonvallot et al. 2018). Rotenone administration in Sprague–Dawley male rats showed decreased GSH levels in the cortex and midbrain (Khurana and Gajbhiye 2013). Dieldrin administration showed reduced levels of GSH in specimen striatal tissue and an increase in oxidative damage following exposure in C57BL/6 J mice (Hatcher et al. 2007). Decreasing levels of GSH in the erythrocytes were observed in ALS patients (Babu et al. 2008). Interestingly, proton magnetic resonance spectroscopy in the motor cortex of ALS patients compared with healthy controls showed decreased levels of GSH (Weiduschat et al. 2014). GSH levels were reduced in studies conducted in the hippocampus and cerebral cortex of Kunming mice, which showed neurodegeneration and cognitive decline (Li et al. 2014). A study conducted in C57BL/6 mice also showed similar results in the hippocampus cerebral cortex in neurodegenerative conditions (Jhoo et al. 2004). Male Wistar albino rats treated with monocrotophos and quinalphos caused a reduction in the levels of GSH (Mishra and Srivastava 2013). In contrast, an increase in the oxidized glutathione levels (GSSG) was observed in the brain specimens (Mishra and Srivastava 2013). Decreased GSH levels are observed in neurodegeneration in patients’ CSF and substantia nigra (Sian et al. 1994; LeWitt et al. 2013). In the early stages of PD, glutathione levels are elevated, suggesting an attempt to protect the brain against the mounting oxidative stress (Rae 2014). Subsequently, ROS accumulation induced by pesticide exposure can lead to a decrease in glutathione synthesis, causing neurodegeneration.

The γ-glutamyl cycle involves 5-oxoproline and is associated with glutathione metabolism and oxidative stress (Cassol et al. 2014). Metabolic profiling of human saliva and urine samples from fifty-two pesticide sprayers predominantly exposed to various pesticides, including profenofos, cypermethrin, endosulfan, kilthion, and pendimethalin, showed upregulation in the metabolite 5-oxoproline (Ch et al. 2019). Increased levels of the metabolites 5-oxoproline were detected in plasma samples of 20 PD patients compared to the control (Trupp et al. 2014). A study investigating the CSF samples of 31 patients with PD revealed an increase in 5-oxoproline levels during the early disease progress (Willkommen et al. 2018).

**Fig. 3:**
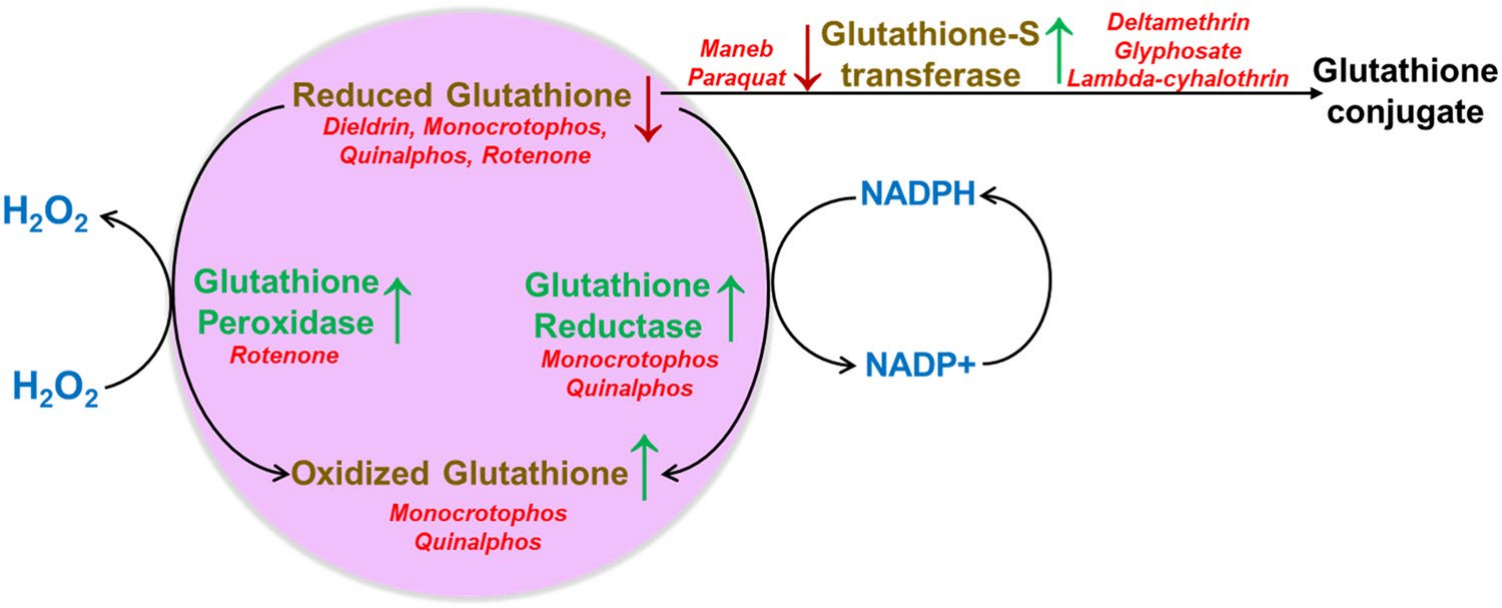
Alterations in the glutathione and glutathione S-transferase levels due to pesticide exposure

Male CFT-Swiss mice, when treated with rotenone, showed an increase in the levels of glutathione peroxidase (GPx) in both the hippocampus and striatum tissue samples (Gokul and Muralidhara 2014). Glutathione reductase (GR), an important antioxidant, showed reduced levels and enzyme activity in the brain specimens of mice treated with a combination of both monocrotophos and quinalphos (Mishra and Srivastava 2013). GPx and GR levels in the blood samples of 50 AD subjects showed statistically lower levels of the metabolites involved in glutathione metabolism (Casado et al. 2008), thus showing that a decrease in the antioxidant enzymes increases oxidative stress leading to neurodegeneration. Hence, oxidative stress can be the common pathological feature between pesticide-induced metabolic rewiring and neurodegeneration. The effect of various pesticides on glutathione metabolism is depicted in Fig. 3.

### Dopamine Metabolism

Tyrosine is an amino acid that produces levodopa (L-DOPA). Formamidine pesticides such as chlordimeform (CDM) and their metabolites have been implicated in the inhibition of monoamine oxidase (MAO) (Beeman and Matsumura 1973). MAO catalyzes the oxidative deamination of neuroactive monoamines like dopamine (DA), serotonin, melatonin, and norepinephrine in the brain (Cho et al. 2021). Inhibition of MAO by CDM is reversible, but its metabolite, N-formyl-4-chloro-o-toluidine (CT), is more potent against MOA (Hollingworth et al. 1979). A study by Hirata and Nagatsu showed that 1 µM rotenone dosage decreased the levels of dopamine (DA) and dopamine metabolites, dihydroxyphenylacetic acid (DOPAC), homovanillic acid (HVA), and noradrenaline (NA) (Hirata and Nagatsu 2005). A study found that the reduced levels of HVA may be related to neurodegeneration and thus a factor in PD (Scatton et al. 1983). Interestingly, organophosphate pesticides such as lebaycid, metacid, and metasystox inhibited MOA and increased catecholamine concentration (Nag and Nandi 1987). Due to affected dopamine metabolism, striatal and cortical HVA levels were reduced. DA is involved in the neuroprotection of dopaminergic neurons, and lower DA levels are involved in neurodegeneration (Segura-Aguilar et al. 2014). The overall DA metabolite level reduction supports the conclusion of DA deficiency in dopaminergic innervation receiving regions (Scatton et al. 1983). The substantia nigra pars compacta displayed reduced dopamine levels in PD patients confirming metabolomic changes in dopamine metabolism leading to neurodegeneration (Gröger et al. 2014).

**Fig. 4:**
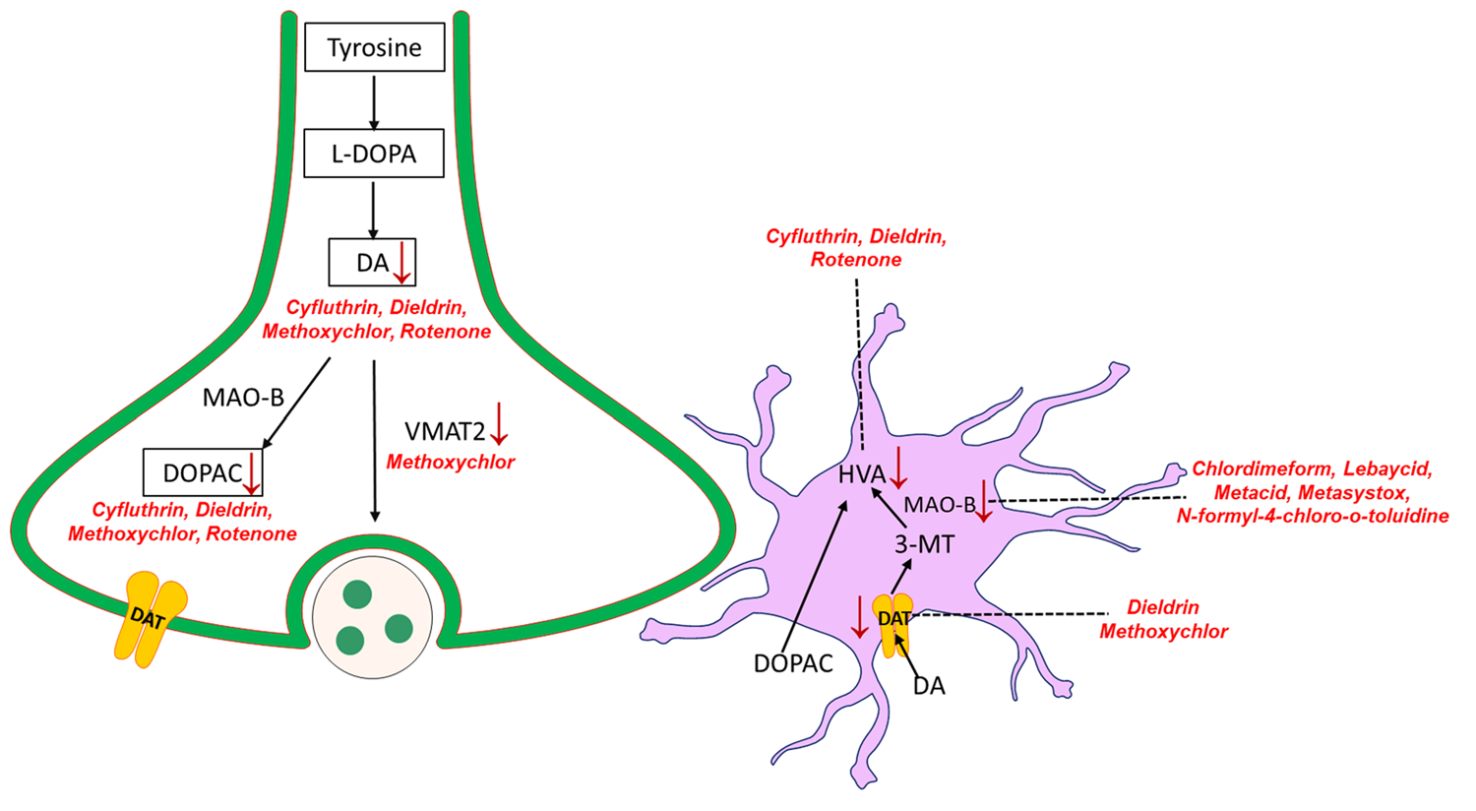
Changes in different metabolite levels involved in dopamine metabolism due to pesticide exposure

Methoxychlor is a synthetically produced organochloride insecticide, and its role in dopamine metabolism alteration was assessed in striatal samples of female CD1 mice where levels of DA, DOPAC, dopamine transporter (DAT), and vesicular monoamine transporter 2 (VMAT2) were reduced (Schuh et al. 2009). Dieldrin treatment in C57BL/6J mice showed decreased dopamine metabolites DA, DAT, DOPAC, and HVA in the mouse cortical tissue specimens (Hatcher et al. 2007). In male Wistar rats, administration of the pyrethroid insecticide cyfluthrin showed a decrease in DA, DOPAC, and HVA metabolites (Rodríguez et al. 2016). Paraquat induced PD *Drosophila* model showed reduced DA and increased DOPAC level (Chaudhuri et al. 2007; Shukla et al. 2014). Furthermore, the reduction in DOPAC levels was also observed in PD patients, indicating their role in the neurodegenerative disorder (Eldrup et al. 1995). Hence, altered metabolites in dopamine metabolism may be a common node between pesticide altered metabolism and neurodegeneration. The effect of various pesticides on dopamine metabolism is depicted in Fig. 4.

### Mitochondrial Dysfunction

Mitochondria are essential in providing cellular energy generated through oxidative phosphorylation. Apart from ROS, metabolic dysregulation has been implicated in causing mitochondrial dysfunction (Moon and Paek 2015). Over the years, studies have shown significant involvement of mitochondrial dysfunction during the pathogenesis of PD (Anandhan et al. 2017b). The intact mitochondria produces ATP and aids in ROS clearance (Moon and Paek 2015). Zebrafish, on exposure to organochlorine pesticides like endosulfan and tetrachlorodibenzo-p-dioxin (TCDD) showed alteration in the tricarboxylic acid pathway metabolites by affecting the oxygen consumption rate (Lee et al. 2020). On exposure to malathion, activity of the enzymes glycogen phosphorylase (GP), phosphoglucomutase (PGM), and hexokinase (HK) was increased, which caused a significant increase in the lactate levels with no changes in the pyruvate levels, thus indicating that it affected cellular respiration in the brain leading to neurodegeneration (Matin et al. 1990). Furthermore, another study supported this observation by showing an increase in HK activity in the brain mitochondria exposed to malathion (Azadbar et al. 2009). Mitochondrial dysfunction, along with changes in other pathways, including creatine phosphate biosynthesis and pyruvate fermentation to lactate, was affected in brain homogenates of mice model mimicking the effects of Gulf War agents (pyridostigmine bromide and permethrin) (Abdullah et al. 2016).

A mass difference enrichment analysis compared 243 masses of control and PD samples and found that metabolites like pyruvate, 2-ketosuccinate, and α-ketoglutarate involved in the TCA cycle were over-represented, causing metabolic dysregulation in PD patients (Willkommen et al. 2018). A study by González-Domínguez et al. showed an increase in the α-ketoglutarate levels causing the alteration of metabolic pathway leading to AD (González-Domínguez et al. 2015). Furthermore, elevated malate levels in plasma samples of PD patients were reported (Trupp et al. 2014). Wu et al. found increased citrate concentrations in CSF samples of 22 PD patients (Wu et al. 2016). However, conflicting results regarding decreased levels of plasma TCA cycle metabolites (citrate, isocitrate, malate, succinate), which were correlated to alteration of pyruvate dehydrogenase activity, were highlighted in a study (Ahmed et al. 2009). Thus, mitochondrial dysfunction due to pesticides is also found in neurodegenerative diseases.

### Energy Metabolism

PD model created by exposure to paraquat was analyzed for any changes in the energy metabolism in dopaminergic cells. The study indicated an increase in glucose, myoinositol, and sedoheptulose concentrations (Lei et al. 2014). A community-based case–control analysis by high-resolution metabolomic assessment was performed for three classes of pesticides indicating that higher organophosphate and organochlorine exposure altered glycolysis and gluconeogenesis metabolites (Yan et al. 2021). Paraquat-treated black mice showed metabolomic alterations in the midbrain and the striatum region. Paraquat decreased metabolites like alanine and lactate within the glycolysis cycle and glutamate within the TCA cycle led to increased citrate levels, increased pAMPK and acetyl-CoA carboxylase (pACC) substrate levels, all of which led to subsequent dopaminergic cell death (Anandhan et al. 2017a). Paraquat also induced changes in energy and pyruvate metabolism in *Drosophila melanogaster* (Shukla et al. 2016).

Metabolomic analysis in mice model mimicking the effects of Gulf War agents showed that several metabolites involved in Krebs cycle like citric acid, malic acid, fumaric acid, succinic acid, and isocitric acid were significantly lowered with respect to control. Furthermore, β-hydroxybutyrate, lactate, glycerol-3-phosphate, and glyceric acid 3-phosphate levels were also lowered (Abdullah et al. 2016). Brain homogenates in dichlorvos-poisoned broilers showed alterations in energy metabolites like acetylcarnitine, dihydroxyacetone phosphate, and glucose-6-phosphate (Huang et al. 2022). Pregnant rats treated with a mixture of pesticides showed decreased levels of adenosine di-phosphate/adenosine monophosphate (ADP/AMP), ATP, lactate, succinate, and aspartate affecting the TCA cycle and energy production (Bonvallot et al. 2018).

On the other hand, metabolites of energy metabolism in CSF samples of 31 PD patients indicated a decrease in the sedoheptulose and an increase in D-glucose-6-sulfate and α-mannosylglycerate (Willkommen et al. 2018). Fructose and mannose are present in increased levels in the CSF of PD patients, and both fructose and mannose metabolisms are involved in the synthesis of α-mannosylglycerate (Trezzi et al. 2017). Glycolysis in PD patients is affected due to the altered level of these metabolites linked to glycolysis (Izumi and Zorumski 2009). Such alterations are influenced by oxidative stress to suppress oxidative phosphorylation in mitochondria (Mazzio and Soliman 2003). A study by Ahmed et al. found that sorbitol concentrations were increased by altering metabolites of the fructose and mannose pathway (Ahmed et al. 2009). Michell et al. observed increased levels of different monosaccharides by analyzing serum metabolites (Michell et al. 2008). Taken together, the loss of energy metabolism leads to persistent dysregulation of neuronal pathways and may lead to neurodegeneration.

### Fatty Acids and Lipid Metabolism

Lipid metabolism is necessary to activate receptors, signal transduction and, modulation for maintaining several biological functions, including cognition (Yadav and Tiwari 2014). Studies on lipid metabolism in neurodegenerative diseases have shown decreased brain cholesterol, galactosylceramide, and sulfatide levels (Colombelli et al. 2015). Rotenone treatment in mice showed alterations in fatty acids linoleic acid, arachidonic acid, and docosahexaenoic acid levels (Tyurina et al. 2015). Furthermore, rotenone also acted with cardiolipins (CLs), increasing CL oxidation metabolites (Tyurina et al. 2015). Polyunsaturated fatty acids CLs were also depleted in rats on exposure to rotenone (Tyurina et al. 2015). Chlorophenotane (DDT) administration in rhesus monkeys also showed alteration in the lipid metabolism metabolites (Sanyal et al. 1986). Another pesticide, paraquat, also altered fatty acid ener metabolism in *Drosophila melanogaster* (Shukla et al. 2016). Furthermore, in brain homogenates of diisopropylfluorophosphate-treated Sprague–Dawley rats, a significant increase in the pro-inflammatory lipid mediators and a small class of anti-inflammatory lipid mediators were seen, indicating the neuroinflammatory response to pesticides (Yang et al. 2019). Metabolites like phosphocholine and glycerophosphocholine of the lipid metabolism were altered in pregnant rats treated with a combination of pesticides (Bonvallot et al. 2018). Metabolomic profiles of the brain of bifenthrin-fed juvenile steelhead trout also showed alteration in lipid metabolism with reduced levels of related metabolites docosahexaenoic acid (DHA) and acetyl-L-carnitine (ALC) (Magnuson et al. 2020).

Increased concentrations of arachidonic acid (ARA), decanoic acid, dihomo-γ-linolenic acid (DGLA), quinic acid, valerenic acid, and 10-hydroxydecanoic acid were noted in PD samples (Willkommen et al. 2018). The increase in ARA and DGLA was correlated to neuroinflammation and oxidative stress, and due to the inflammatory process, the release of ARA from membranes was higher. DGLA is a PUFA that can form pro-inflammatory ARA and is released before the anti-inflammatory compounds (Bazinet and Laye 2014). Increased ARA metabolism due to oxidative stress and neuroinflammation caused neurodegeneration (Bosetti 2007). PUFA depletion has also been indicated to cause mitochondrial dysfunction contributing to PD pathogenesis (Tyurina et al. 2015). Furthermore, the role of the fatty acid metabolism in CNS disorders and the diagnostic and therapeutic potential of metabolomics data were reviewed by Bogie et al. (Bogie et al. 2020). Many pesticides affect more than one metabolic pathway; for example, imidacloprid altered six metabolic pathways in mice hippocampus, including lipid metabolism, amino acid metabolism, nucleotide metabolism, carbohydrate metabolism, energy metabolism, and metabolism of cofactors and vitamins (Zheng et al. 2020).

The summary of various pesticides and the selected metabolic pathways affected by their exposure is listed in Table 1.

**Table 1:** Studies on the pesticide-induced changes in the metabolites in the nervous tissue

Pesticides	Model/cells—sample type	Pathway affected	Changes induced	References
**In vitro studies**				
Paraquat	SK-N-SH cell line	Pentose phosphate pathway	Glucose↑, myoinositol↑, sedoheptulose↑	Lei et al. (2014)
**In vivo studies**				
Paraquat	Oregon R + (wild-type) *Drosophila* flies	Fatty acid and amino acid metabolism	Glycerolipid, inositol phosphate, glycerophospholipid, fatty acid ↑ Proline, arginine, valine, aspartate, alanine, glutamate, leucine, isoleucine ↓	Shukla et al. (2016)
Endosulfan and tetrachlorodibenzo-p-dioxin	Zebrafish	Tricarboxylic acid pathway	OCR↓	Lee et al. (2020)
Chlorfenapyr	Zebrafish	Amino acid metabolism	Alanine, tyrosine, lysine, leucine, phenylalanine, and valine ↑ 7-Methylxanthine and taurine ↓	Chen et al. (2021)
Dieldrin	C57BL/6 J mice striatal tissue	Glutathione pathway	GSH↓	Hatcher et al. (2007)
Dieldrin	C57BL/6 J mice striatal tissue	Dopamine pathway	DA↓, DOPAC↓, DAT↓, HVA↓	Hatcher et al. (2007)
Maneb	Male Swiss albino mice brain samples	Glutathione pathway	GST↑	Singhal et al. (2011)
Methoxychlor	female CD1 mice striatal samples	Dopamine pathway	DA↓, DOPAC↓, DAT↓, VMAT2↓	Schuh et al. (2009)
Rotenone	CFT-Swiss mice hippocampus and striatum samples	Glutathione pathway	GPx↑	Gokul and Muralidhara (2014)
N-Formyl-4-chloro-o-toluidine	Male Swiss white mice	Dopamine pathway	MAO↓	Hollingworth et al. (1979)
Paraquat	Male Swiss albino mice brain samples	Glutathione pathway	GST↑	Singhal et al. (2011)
Paraquat	C57BL/6 J mice	Glycolysis and TCA cycle	Citrate, pAMPK, and acetyl-CoA carboxylase ↑ Alanine, lactate and glutamate ↓	Anandhan et al. (2017a)
Permethrin	C57BL6 mice	TCA cycle, Glycolysis and energy cycle	Citric acid, malic acid, fumaric acid, succinic acid, and isocitric acid ↓ β-hydroxybutyrate, lactate, glycerol-3-phosphate, and glyceric acid 3-phosphate ↓	Abdullah et al. (2016)
Cyfluthrin	Male Wistar rats	Dopamine pathway	DA↓, DOPAC↓, HVA↓	Rodríguez et al. (2016)
Deltamethrin	Adult male Wistar rat cortical tissue	L-Kynurenine pathway	KYNA↓	Zielińska et al. (2005)
Fenpropathrin	Adult male Wistar rat cortical tissue	L-Kynurenine pathway	KYNA↓	Zielińska et al. (2005)
Mixture of acetochlor, bromoxynil, carbofuran, chlormequat, ethephon, fenpropimorph, glyphosate, imidacloprid	Pregnant Wistar rats	TCA cycle, lipid and amino acid metabolism	Lysine, n-acetylaspartate, inosine, ethanolamine, and oxidized glutathione ↑ Lipids, aspartate, lactate, glutamine, succinate, serine, phosphocholine, glycerophosphocholine, urine, ADP/AMP, and ATP ↓	Bonvallot et al. (2018)
Monocrotophos	Wistar albino male adult rats brain samples	Glutathione pathway	GSH↓, GSSG↑	Mishra and Srivastava (2013)
Monocrotophos and quinalphos	Wistar albino male adult rats brain samples	Glutathione pathway	GR↑	Mishra and Srivastava (2013)
Quinalphos	Wistar albino male adult rats brain samples	Glutathione pathway	GSH↓, GSSG↑	Mishra and Srivastava (2013)
Rotenone	Male Wistar rat striatal tissue	Dopamine pathway	DA↓, DOPAC↓, HVA↓, NA↓	Hirata and Nagatsu (2005)
Lebaycid, metacid, and metasystox	Male albino rat brain mitochondria	Dopamine pathway	MAO↓	Nag and Nandi (1987)
Malathion	Adult female albino rat brain	Mitochondrial dysfunction	Lactate↑	Matin et al. (1990)
Diazinon	Swiss-Webster male mice plasma	L-Kynurenine pathway	QA↓, KYNA↓, KYN↑	Seifert and Pewnim (1992)
Rotenone	Male Lewis rats	Fatty acid metabolism	Linoleic acid↓, arachidonic acid↓, docosahexaenoic acid↓	Tyurina et al. (2015)
Rotenone	Male Lewis rats	Mitochondrial signaling pathway	PUFA CLs ↓	Tyurina et al. (2015)
Rotenone	Sprague–Dawley male rat cortex and midbrain	Glutathione pathway	GSH↓	Khurana and Gajbhiye (2013)
Dichlorvos	Broilers	Energy and amino acid and nucleic acid metabolism	Dihydroxyacetone phosphate, glucose 6-phosphate ↑, acetylcarnitine ↓ Gamma-glutamylcysteine, glutathione disulfide, dipeptide compound ↑ Uridine ↓ Inosine 5′-monophosphate, hypoxanthine, uridine 5′-monophosphate ↑	Huang et al. (2022)
**Human studies**				
Deltamethrin	Human blood	Glutathione pathway	GST↓	Diken et al. (2017)
Glyphosate	Human blood	Glutathione pathway	GST↓	Diken et al. (2017)
Lambda-cyhalothrin	Human blood	Glutathione pathway	GST↓	Diken et al. (2017)
Cypermethrin, endosulfan, kildor, kilthion, pendimethalin, and profenofos	Pesticide sprayers saliva and urine samples	Glutathione pathway	5-Oxoproline↑	Ch et al. (2019)

*DA* dopamine, *DAT* dopamine transporter, *DOPAC* dihydroxyphenylacetic acid, *GSH* reduced glutathione, *GR* glutathione reductase, *GSSG* oxidized glutathione, *GST* glutathione S-transferase, *HVA* homovanillic acid, *KYN* kynurenine, *KYNA* kynurenic acid, *MAO* monoamine oxidase, *NA* noradrenaline, *OCR* oxygen consumption rate, *PUFA CLs* polyunsaturated fatty acids cardiolipins, *QA* quinolinic acid, *SK-N-SH* human dopaminergic neuroblastoma cell line, *VMAT2* vesicular monoamine transporter 2

## Discussion and Conclusion

The review summarizes the epidemiological and experimental studies looking for the correlation between metabolites altered by pesticide exposure and their link with neurodegenerative disorders. Reports indicate that less than 10% of PD cases are purely genetics (Simon et al. 2020).However, environmental factors play a significant role, which acts independently or combined with genetic factors (Caudle et al. 2011). Furthermore, aging is detrimental to neurodegenerative disorders due to increased and accumulated toxicant exposure which can aggravate symptoms of neurodegeneration. The epidemiological studies identify pesticide exposure as an essential cause of neurodegenerative diseases (Freire and Koifman 2012; Moretto and Colosio 2013). However, the mechanisms and the metabolites involved in pesticide-induced neurodegeneration are poorly explored.

Neurodegenerative disorders have previously been associated with metabolic abnormalities. Hence, the metabolomic approach is a promising tool that helps identify abnormal metabolite levels and pathways associated with major neurodegenerative diseases (Cai et al. 2012). Therefore, metabolomic studies will be helpful in identifying the abnormal levels of metabolites that are indicative of specific underlying disease processes and aid in the management of neurodegenerative diseases. In addition, the differentially altered metabolites can then act as biomarkers for the early detection of neurodegenerative disorders and could help counter damage caused (Patti et al. 2012).

Inflammation-related pathways like tryptophan catabolism, arachidonic acid metabolism, and histidine metabolism, when altered by different pesticides, lead to the accumulation of neurotoxic products driving towards neurodegeneration. Pesticide exposure-induced oxidative stress can cause mitochondrial dysfunction wherein the use of organochlorines and organophosphates has shown alteration of mitochondrial energy metabolism pathways. Mitochondrial dysfunction is often accompanied by oxidative stress and impaired cellular respiration and also leads to apoptosis/autophagy, which is often some of the symptoms of neurodegenerative disorders. Samples from the brain tissue, CSF, serum, blood, saliva, and urine samples all have shown changes in the various metabolite levels. The role of altered dopamine metabolism in Parkinson’s has been well established. Organochlorine pesticides have been shown to affect dopamine metabolism, which could hamper neurotransmission and dopaminergic neuronal survival as a consequence of pesticide resultant oxidative stress. Furthermore, pesticides alter glutathione synthesis and metabolism, further exacerbating the production of reactive oxygen species, thus displaying their role in inducing neurodegenerative disorders. The brain and the activities of the resident neurons are energy driven, meaning they depend on mitochondria for a continuous supply of energy for their abundant activities. The majority of the metabolomic studies have shown that along with mitochondrial dysfunction, the TCA cycle, which is the source of ATP for the cellular activities of these neurons, is impaired due to variations in the metabolite levels within the cycle. Studies show that glycolysis is similarly altered in PD patients, which exhibits another parallel between the pesticide effects and neurodegenerative symptoms. Studies also reveal the alteration of several pro- and anti-inflammatory mediators upon pesticide exposure that are recruited as part of the immediate immune response of the cell. Most importantly, amino acid metabolism, which serves a key role as precursors or intermediates in several overlapping metabolic pathways, like purine and pyrimidine synthesis, neurotransmitter metabolism, citric acid cycle, and urea biosynthesis, is also affected. Altered levels of various amino acids serve as essential early biomarkers for various diseases like Alzheimer’s and PD, although when it comes to human studies, there are several cofactors like the diet that may affect the levels of the metabolites within an individual. However, these pathway changes often culminate in neuronal death, a primary factor for the onset of neurodegenerative diseases.

Therefore, this review summarizes the molecular pathways that are vulnerable to chronic pesticide toxicity to understand their physiology, leading to better prediction of pesticide-related health outcomes. Metabolomics data focusing on the nervous system are still scarce though the available evidence supports the existence of specific mechanistic associations between neuronal damage and pesticide exposure leading to neurodegeneration. However, it is also important to validate how the changes in the individual metabolite associated with a particular neurodegenerative phenotype have any measurable influence on the phenotype. A further combined approach of different omics will help better understand the physiological and pathologic processes involved in the human brain.

## Data Availability

Not applicable.
